# A Mathematical Model of Intra-Colony Spread of American Foulbrood in European Honeybees (*Apis mellifera* L.)

**DOI:** 10.1371/journal.pone.0143805

**Published:** 2015-12-16

**Authors:** Eduardo O. Jatulan, Jomar F. Rabajante, Charina Gracia B. Banaay, Alejandro C. Fajardo, Editha C. Jose

**Affiliations:** 1 Institute of Mathematical Sciences and Physics, University of the Philippines Los Baños, Laguna, Philippines; 2 Institute of Biological Sciences, University of the Philippines Los Baños, Laguna, Philippines; 3 UPLB Bee Program, University of the Philippines Los Baños, Laguna, Philippines; University of Arizona, UNITED STATES

## Abstract

American foulbrood (AFB) is one of the severe infectious diseases of European honeybees (*Apis mellifera* L.) and other *Apis* species. This disease is caused by a gram-positive, spore-forming bacterium *Paenibacillus larvae*. In this paper, a compartmental (SI framework) model is constructed to represent the spread of AFB within a colony. The model is analyzed to determine the long-term fate of the colony once exposed to AFB spores. It was found out that without effective and efficient treatment, AFB infection eventually leads to colony collapse. Furthermore, infection thresholds were predicted based on the stability of the equilibrium states. The number of infected cell combs is one of the factors that drive disease spread. Our results can be used to forecast the transmission timeline of AFB infection and to evaluate the control strategies for minimizing a possible epidemic.

## Introduction

American Foulbrood (AFB) is considered as the most widespread and destructive of the bee brood diseases [1]. It is considered to be very infectious once clinical symptoms are visible, that is, infected colonies are likely to succumb to the disease if left untreated [2]. AFB is spreading around the world and is causing an alarm to stakeholders in the bee industry. In the Philippines alone, according to the survey done by Cervancia et al. [3], out of 139 apiaries 46% were found to be infected by AFB.

AFB is caused by the gram-positive, spore-forming bacterium *Paenibacillus larvae* [1]. The bacterium exists in two forms–the spore stage and the vegetative stage [4]. AFB is only contagious during the spore stage of the bacterium [5]. The spores infect the young brood (larva) of the colony which may cause death to the larva. In particular, larvae that are 12–36 hours old are the most susceptible to the disease [6]. The infected larvae will die after their cells are sealed. The cell with deceased larvae contains millions of spores that are viable for many years. These spores can contaminate the cell comb, honey storage, and the materials in beekeeping. Moreover, these spores are resistant to extreme temperature and to most anti-bacterial agents. The pathogen does not affect the adult bees but the adults can be carriers of spores by cleaning the surroundings of a contaminated cell and by robbing from an infected colony or from contact with spore infected flora.

There are two different modes of AFB spores transmission–horizontal and vertical. Horizontal transmission refers to the AFB spores transmission between the individuals within and between colonies of honeybees while vertical transmission refers to the AFB spores transmission from a mother colony to a daughter swarm. For the demonstration of colony level vertical disease transmission, one can see the paper by Fries et al. [7]. Horizontal transmission is more virulent compared to the vertical transmission [8]. Horizontal transmission can be divided into intra-colony (within colony) and inter-colony (between colonies) transmission. In this paper, we are concerned with the intra-colony horizontal transmission of AFB disease.

Robbing, the phenomenon where honeybees steal honey from a colony infected with AFB, is considered to be one of the main routes of horizontal transmission of AFB under natural conditions [8]. Robbing frequently happens when there is scarcity of food and when colonies are weak. Aside from robbing, drifting can also spread AFB spores between colonies. Drifting is the case where the worker of one colony enters another hive by mistake. However, drifting of bees is of minor importance in generating clinical cases [9]. Intra-colony transmission of spores can be significantly influenced by honeybee behavior such as the movement of infected honey stores and indirect bee-to-bee contact within the colony [8].

Mathematical models have been used to study epidemics of bee diseases. Datta et al. [10] mathematically investigated the inter-colony spread of AFB in honeybees using the data gathered from the island of Jersey, USA. They showed that distance and owner-based transmissions significantly contributed to the spread of AFB. Gavina et al. [11] proposed a solution to this problem using a mathematical program. They formulated a mathematical program to optimally distribute the location of beehives in a bee farm to minimize the spread of diseases among different colonies.

Here, we formulated and analyzed a mathematical model of intra-colony spread of AFB in a controlled environment where there is enough resources that the colony can forage. The occurrence of robbing could be negligible. We assumed that the foraging area in the controlled environment is initially contaminated by AFB spores and there is a constant rate of contamination. The aim of this model is to elucidate the long term effect of the exposure of the colony to AFB. A compartmental ordinary differential equations (ODE) model is used to describe the dynamics of the disease spread. In the development of our model, we also considered the queen’s laying rate and the cleaning activity of the house bees. We used qualitative analytic approach and numerical simulations to determine the stability of the equilibrium states of the ODE model. We also determined the factor that serves as the catalyst of the spread of the disease. The results of the analysis of this model could help identify critical control points where AFB epidemics can be slowed down or stopped at its early stage.

## Methods

### Main Assumptions

In the formulation of the model, it is assumed that colonies are under controlled environment where there is no scarcity of food. The case of robbing of food by a forager can be disregarded. The probability that forager bees from the healthy colony collect from a contaminated food is constant.

Furthermore, it is assumed that a brood can become infected when it is fed by a spore-carrying hive bee and when it is laid in an infected cell comb. The number of infected house (adult) bees in a colony is directly proportional to the number of spores present in the nectar/honey storage area. The number of eggs that will be lain by the queen depends on the resources available and on the number of house bees and forager bees present in the hive to rear the brood. An adult bee is a spore-carrier when it is carrying enough number of spores, outside or inside its body, capable of transmitting AFB within the colony.

### Mathematical Model

**Fig 1:**
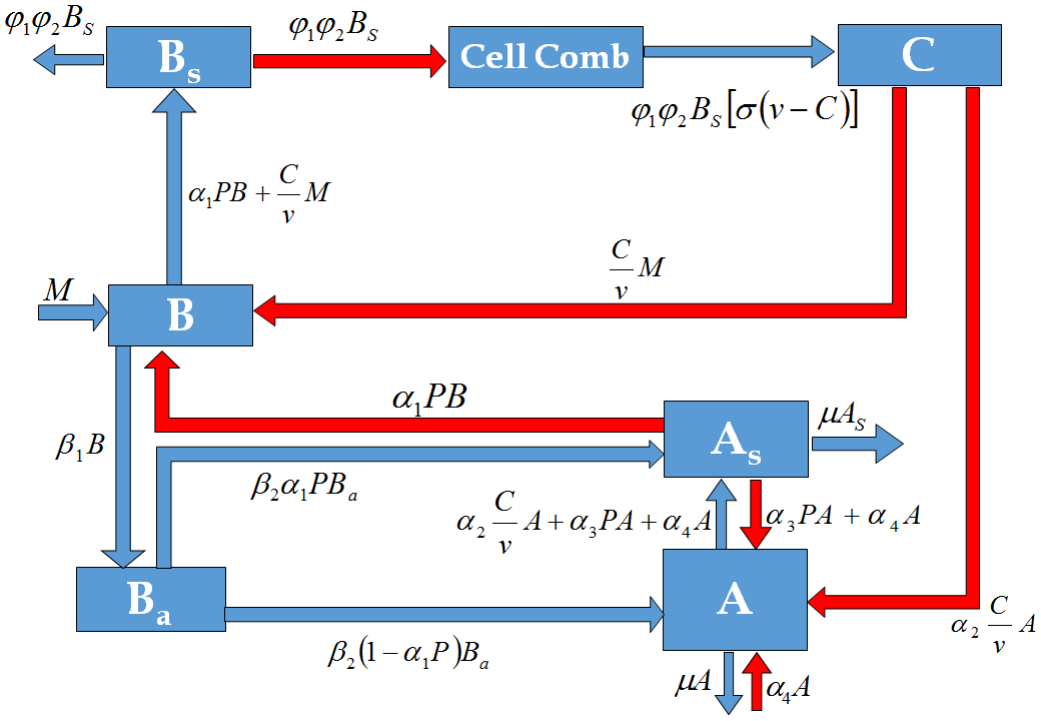
The compartmental diagram of the interaction of bees and the spread of AFB spores within a colony. The red arrows indicate infection while the blue arrows indicate transition of states (refer to Table 1 for the definition of the state variables).

We considered a compartment model wherein every population is divided into states, and an individual of a population transfers from one state to another with a suitable rate (Fig 1). Here, we do not differentiate the house bees from forager bees, that is, we simply treated them as adult bees. This is based on the fact that bees inside the hive interact constantly with each other. The status of house bees is almost the same as that of the forager bees.

In the model, we considered 6 state variables (Table 1) with parameters summarized in Table 2. We used the Susceptible-Infectious (SI) framework for the spread of AFB in broods and adult bees. The mathematical model of the spread of AFB in a controlled environment (Fig 1) is given by the following system:

$$\begin{array}{c} \frac{dB}{dt}=M-\alpha_{1}PB-\frac{C}{v}M-\beta_{1}B \end{array}$$ (1)

$$\begin{array}{c} \frac{dB_{a}}{dt}=\beta_{1}B-\beta_{2}B_{a} \end{array}$$ (2)

$$\begin{array}{c} \frac{dB_{s}}{dt}=\alpha_{1}PB+\frac{C}{v}M-\varphi_{1}\varphi_{2}B_{s} \end{array}$$ (3)

$$\begin{array}{c} \frac{dC}{dt}=\varphi_{1}\varphi_{2}B_{s}[\sigma (v-C)] \end{array}$$ (4)

$$\begin{array}{c} \frac{dA}{dt}=\beta_{2}(1-\alpha_{1}P)B_{a}-\alpha_{2}\frac{C}{v}A-\alpha_{3}PA-\alpha_{4}A-\mu A \end{array}$$ (5)

$$\begin{array}{c} \frac{dA_{s}}{dt}=\beta_{2}\alpha_{1}PB_{a}+\alpha_{2}\frac{C}{v}A+\alpha_{3}PA+\alpha_{4}A-\mu A_{s} \end{array}$$ (6)

where $P=\frac{A_{s}}{1+A+A_{s}}$ and $M=L\frac{A+A_{s}}{w+A+A_{s}}$.

For simplicity, we write the system as

$$\begin{array}{c} \frac{dX_{i}}{dt}=F_{i}\left(X\right),i=1,2,\cdots ,6 \end{array}$$ (7)

where *X* = (*X*_1_, *X*_2_, *X*_3_, *X*_4_, *X*_5_, *X*_6_) = (*B*, *B*_*a*_, *B*_*s*_, *C*, *A*, *A*_*s*_).

**Table 1:** Description of the state variables.

Variables	Description
*B*	The number of healthy brood in the hive before *n* hours old (*n* depends on the susceptibility of bees)
*B*_*a*_	The number of healthy brood in the hive beyond *n* hours old
*B*_*s*_	The number of infected brood in the hive
*C*	The number of infected cell combs (due to diseased dead brood)
*A*	The number of adult bees without AFB spores
*A*_*s*_	The number of adult bees with AFB spores

**Table 2:** Parameters used in the model.

Parameters	Description
*L*	The queen’s maximum laying rate per day
*w*	A coefficient that influences the rate at which the term *M*(see main text for the definition of *M*) approaches *L* as *A* + *A*_*s*_ gets large
*β*_1_	The rate at which broods become immune with the spores
*β*_2_	The rate at which immune broods become adult bees
*α*_1_	The rate at which broods are infected by spore-carrier adult bees
*φ*_1_	The death rate of infected broods
*φ*_2_	The rate at which a diseased dead brood is cleaned by adult bees
*v*	The maximum number of cells in the colony
*σ*	The percentage of clean cell combs that will be contaminated by AFB spores
*α*_2_	The rate at which adult bees become infected by the infected cell
*α*_3_	The rate at which adult bees become infected upon contact with spore-carrier adult bees
*α*_4_	The rate at which adult bees become infected upon contact with the foraging (food source) area
*μ*	The death rate of adult bees (both spore-carrier and spore-free)

The term $M=L\frac{A+A_{s}}{w+A+A_{s}}$ in Eq (1) is the number of eggs that a queen can produce per day. This term is known to be affected by the number of adult bees in the colony. The parameter *L* is the maximum queen’s laying rate in a day. The factor $\frac{A+A_{s}}{w+A+A_{s}}$ describes how the queen’s laying rate is affected by the number of adult bees present in the hive. As the total number of adult bees *A* + *A*_*s*_ increases, the queen’s laying rate approaches its maximum [12]. Moreover, *w* determines the rate at which *M* approaches *L* as the number of adult bees gets large.

The term *α*_1_ *PB* in Eqs (1) and (3) is the number of broods that become infected per day due to their contact with spore-carrier adult bees, where $P=\frac{A_{s}}{1+A+A_{s}}$. This case usually happens when a spore-carrier adult bee feeds the broods. *P* here is the probability that a brood will be in contact with spore-carrier adult bees. Moreover, *PB* is the number of broods that can be infected by spore-carrier adult bees per day.

The term $\frac{C}{v}M$ in Eqs (1) and (3) is the number of broods that become infected when they are laid in infected cells per day. The expression $\frac{C}{v}$ is the probability that a brood will be laid in an infected cell.

The term *β*_1_ *B* in Eqs (1) and (2) is the number of young broods that become resistant to AFB spores. The parameter *β*_1_ is the rate at which broods become resistant to AFB spores. This means that the broods will survive even if they carry spores. However, immunity to spores does not make the broods spore-free (i.e., they can be spore-carriers).

The term *β*_2_ *B*_*a*_ in Eq (2) is the number of broods that become adult bees per day. The parameter *β*_2_ is the rate at which broods become adult bees, also called eclosion rate. The broods that survived AFB may carry spores when they become adult bees.

The term *φ*_1_ *B*_*s*_ in Eq (3) is the number of broods that die because of AFB spores infection. The parameter *φ*_1_ is the death rate of infected broods where 1/*φ*_2_ is the average number of days for adult bees to clean the diseased dead brood.

The term *φ*_1_ *φ*_2_ *B*_*s*_ [*σ*(*v* − *C*)] in Eq (4) is the number of cell combs that can be contaminated (due to diseased dead brood) by adult bees upon cleaning/removal of the diseased dead brood. The factor *φ*_1_ *φ*_2_ *B*_*s*_, which we mentioned above, can also be considered as the number of cells that become infected once an infected brood dies. As the adult bees clean the cells with the diseased dead brood, they have the tendency to spread the spores in other cell combs. We assumed that the percentage of the non-contaminated cell combs that can be contaminated by this process is given by a constant parameter *σ*. Hence, the factor *σ*(*v* − *C*) is the number of cell combs that can be contaminated for every removal of one diseased dead brood. The expression *v* − *C* tells us that the number of infected cell combs cannot be more than the number of cell combs in the colony. If *C* reaches the maximum number of cell combs *v*, *φ*_1_ *φ*_2_ *B*_*s*_ [*σ*(*v* − *C*)] becomes zero.

The broods that survive AFB spore infection do not automatically become spore-free when they become adult bees [13]. As broods become adult bees, they can be spore-carriers. We set *β*_2_ *α*_1_ *PBa* in Eq (6) as the number of broods that become spore-carrier adult bees after being resistant. The term *β*_2_(1 − *α*_1_ *P*)*B*_*a*_ in Eq (5) is the number of broods that become spore-free adult bees per day.

The term $\alpha_{2}\frac{C}{v}A$ in Eqs (5) and (6) is the number of adult bees that become spore-carriers upon contact with the infected cells. Here, $\frac{C}{v}$ is the probability that a spore-free adult bee will be in contact with an infected cell.

The term $\alpha_{3}\frac{A_{s}}{1+A+A_{s}}A$ in Eqs (5) and (6) is the number of spore-free adult bees that become spore-carriers upon contact with the spore-carrier adult bees. From the main assumptions, the number of spore-carrier bees has an indirect effect on the spread of spores in the storage area. The parameters involved in the indirect effect of spore-carriers on the spread of spores in the storage area are embedded in the term $\alpha_{3}\frac{A_{s}}{1+A+A_{s}}A$. On the other hand, *α*_4_ *A* is the number of spore-free adult bees that become spore-carriers upon contact with the contaminated food outside the hive in the controlled environment. We assumed here that the rate of infection *α*_4_ is constant. Lastly, *μ* is the collective death rate of the spore-free and spore-carrier adult bees.

## Results

The ODE system Eqs (1)–(6) is shown to have a unique solution using the Existence–Uniqueness Theorem [14]. The solutions to the model are eventually confined in an invariant set

$$\begin{array}{ccc} \Gamma & = & \{(B,B_{a},B_{s},C,A,A_{s})|0\leq B+B_{s}\leq \frac{L}{\gamma },0\leq B_{a}\leq \frac{L+\beta_{1}B_{0}}{\beta_{2}},\\ & & \;0\leq C\leq v,0\leq A+A_{s}\leq \frac{L+\beta_{1}B_{0}+\beta_{2}B_{a0}}{\mu }\}. \end{array}$$

This means that the possible maximum number of the adult bees is $\frac{L+\beta_{1}B_{0}+\beta_{2}B_{a0}}{\mu }$ while the possible maximum number of broods is $\frac{L}{\gamma }+\frac{L+\beta_{1}B_{0}}{\beta_{2}}$, where *B*_0_, *B*_*a*0_ are initial values of *B* and *B*_*a*_, respectively. The model was analyzed using the standard linear stability analysis [14, 15] and numerical simulations.

The model has three possible equilibrium states (see S1 File): the trivial (0,0,0,0,0,0), spore-free equilibrium state $\left(\frac{L-w\mu }{\beta_{1}},\frac{L-w\mu }{\beta_{2}},0,0,\frac{L-w\mu }{\mu },0\right)$, and the extinction (colony collapse) equilibrium state (0,0,0, C*,0,0), where 0 < C* ≤ *v*. It was also found out that the trivial and spore-free equilibrium states exist if *α*_4_ = 0. Moreover, the trivial equilibrium state and the spore-free equilibrium state are locally asymptotically stable if $\frac{L}{\mu w}>1$ and $\frac{L}{\mu w}<1$, respectively. On the other hand, if the value of *α*_4_ ≠ 0 then the system has only one equilibrium state, the extinction equilibrium state.

### The case when there are no spores from the foraging area (*α*_4_ = 0)

**Fig 2:**
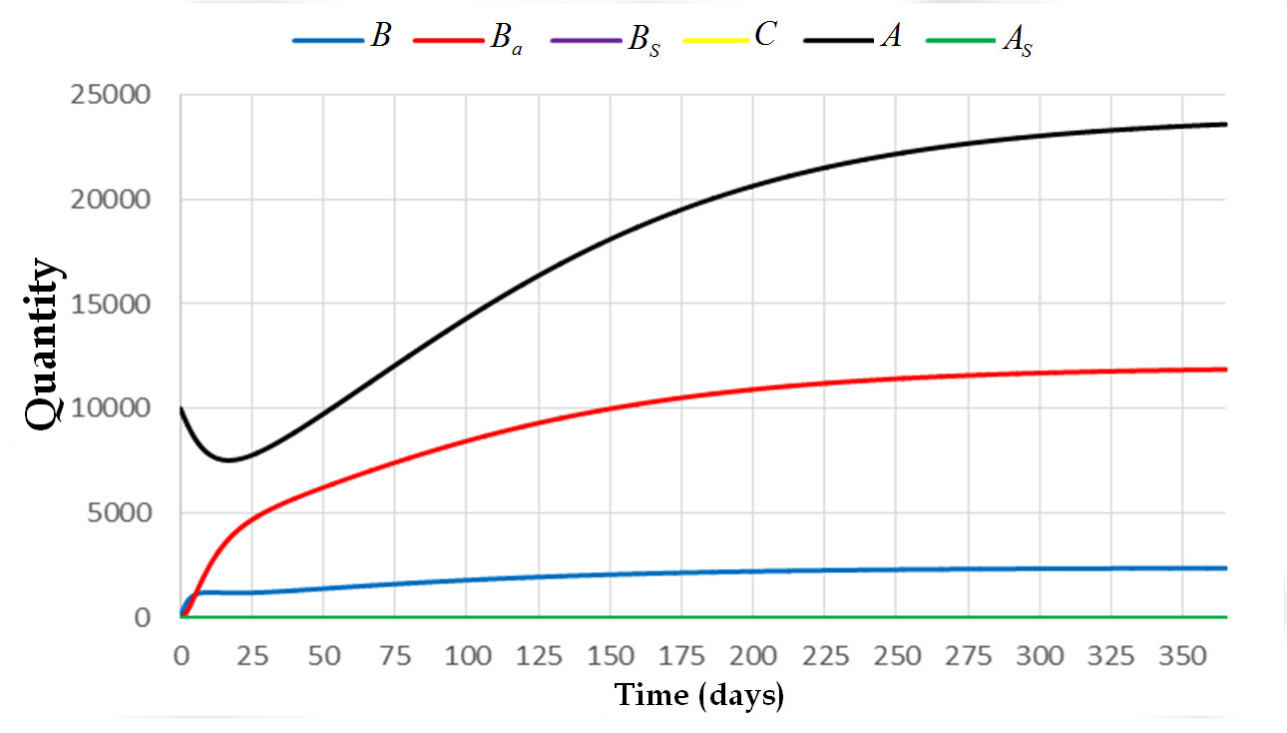
The trajectory of the system Eq (7) with initial condition (0,0,0,0,10000,0) and parameters *L* = 1500, *w* = 21000, *v* = 10000, *α*_4_ = 0, *β*_1_ = 1/3, *β*_2_ = 1/15, and *μ* = 1/30. This figure shows that the colony survives if the value of $\frac{L}{w\mu }>1$ and *α*_4_ = 0.

**Fig 3:**
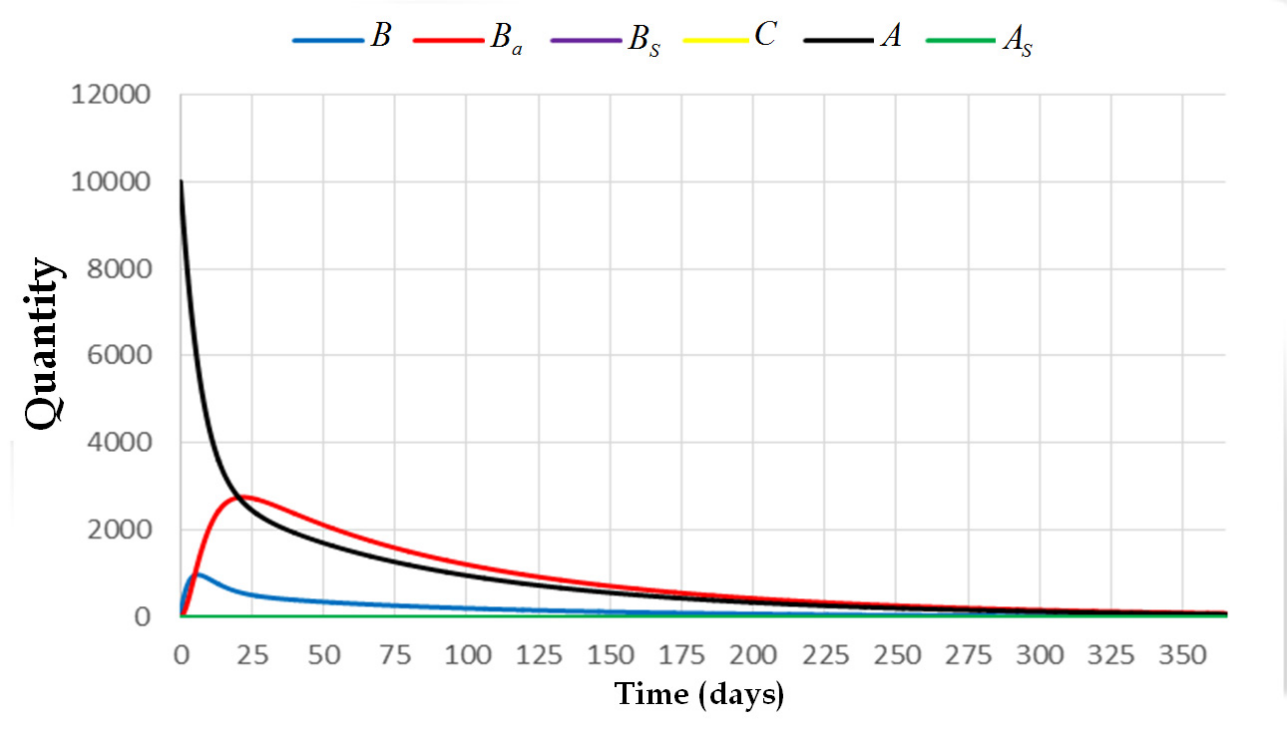
The trajectory of the system Eq (7) with initial condition (0,0,0,0,10000,0) and parameters *L* = 1500, *w* = 21000, *v* = 10000, *α*_4_ = 0, *β*_1_ = 1/5, *β*_2_ = 1/13, and *μ* = 2/21. This figure shows that the colony dies out if the value of $\frac{L}{w\mu }>1$ and *α*_4_ = 0.

Fig 2 illustrates what would happen to the system if *α*_4_ = 0. As we can see in the figure, the solutions of *B*, *B*_*a*_ and *A* stabilize at $\frac{L-\mu w}{\beta_{1}}=2400$, $\frac{L-\mu w}{\beta_{2}}=12000$ and $\frac{L-\mu w}{\mu }=24000$, respectively. In this figure, we used the following parameters: $w=21000,L=1500,\text{and}\;\mu =\frac{1}{30}$ that make $\frac{L}{w\mu }<1$. For the same values of *w* and *L*, if we increase the death rate of adult bees *μ* to 2/21, then $\frac{L}{w\mu }>1$. Thus, the colony is expected to die. This is expected to happen since there are many adult bees that die per day. Hence, the queen will lay less number of eggs since there is not enough number of adult bees that will nurse the broods or will forage for food. Fig 3 shows that the solution of the system converges to the trivial equilibrium state if the value of $\frac{L}{w\mu }$ is greater than one.

**Fig 4:**
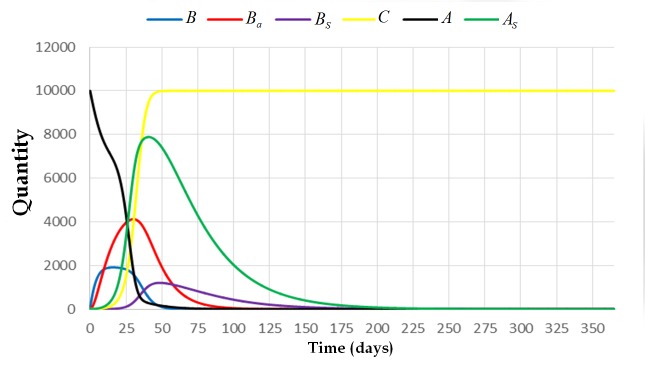
The trajectory of the system Eq (7) with initial condition (0,0,1,0,10000,0) and parameters *L* = 1500, *w* = 21000, *v* = 10000, *α*_4_ = 0, *α*_3_ = 0.0005, *α*_2_ = 1, *α*_1_ = 0.001, *φ*_1_ = 1/3, *φ*_2_ = 1, *σ* = 0.001, *β*_1_ = 1/5, *β*_2_ = 1/13, and *μ* = 1/30. This figure shows that the colony dies out if one of the broods is already infected. The solution converges to the extinction equilibrium state.

**Fig 5:**
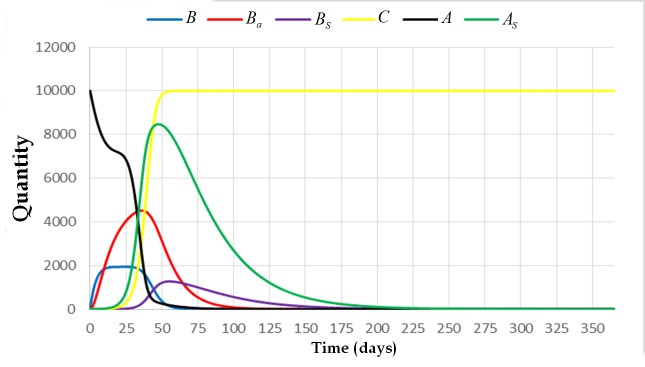
The trajectory of the system Eq (7) with initial condition (0,0,0,1,10000,0) and parameters *L* = 1500, *w* = 21000, *v* = 10000, *α*_4_ = 0, *α*_3_ = 0.0005, *α*_2_ = 1, *α*_1_ = 0.001, *φ*_1_ = 1/3, *φ*_2_ = 1, *σ* = 0.001, *β*_1_ = 1/5, *β*_2_ = 1/13, and *μ* = 1/30. This figure shows that the colony dies out if one of the cell combs is already contaminated with spores.

**Fig 6:**
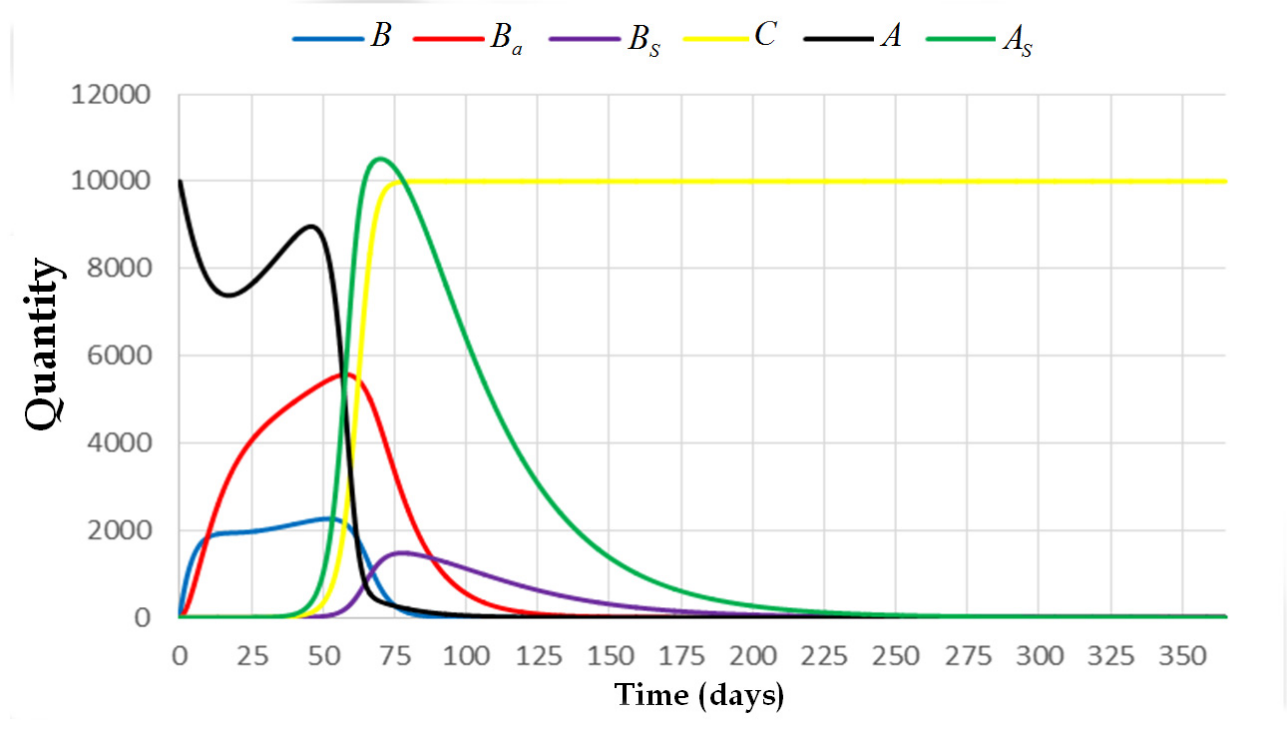
The trajectory of the system Eq (7) with initial condition (0,0,0,0,10000,1) and parameters *L* = 1500, *w* = 21000, *v* = 10000, *α*_4_ = 0, *α*_3_ = 0.0005, *α*_2_ = 1, *α*_1_ = 0.001, *φ*_1_ = 1/3, *φ*_2_ = 1, *σ* = 0.001, *β*_1_ = 1/5, *β*_2_ = 1/13, and *μ* = 1/30. This figure shows that the colony dies out if one of the adult bees is carrying enough spores to start infection.

The stability of the trivial equilibrium state and the spore-free equilibrium state depends on the given initial condition (see S1 File). In particular, if the value of either *B*_*s*_, *C*, or *A*_*s*_ is nonzero then the two equilibrium states are not stable. From this, we say that the domain of attraction of the spore-free equilibrium state is given by the set $\left\{\left(B,Ba,0,0,A,0\right)|B,Ba,A\in R^{⊕}\right\}$. This result implies that if the spores have entered the colony, by any means, the colony will eventually die or collapse. As an illustration of this claim, in Figs 4, 5 and 6, we set the initial condition of *B*_*s*_ = 1, *C* = 1, and *A*_*s*_ = 1, respectively. From the simulations we can see that the solutions to the system do converge to (0,0,0, C*,0,0). Hence, existence of one infected brood or one spore-carrier adult bee is sufficient to start the AFB epidemics within the colony.

As the spores enter the colony, the colony will eventually die or collapse. This has been observed from the results where at least one of the following is nonzero: the rate at which adult bees become infected upon contact with the infected foraging area (*α*_4_), initial number of the infected brood in the hive (*B*_*s*0_), initial number of infected cell combs due to diseased dead brood (*C*_0_), and the initial number of spore–carrying adult bees (*A*_*s*0_).

We can also observe from Figs 5 and 6 that there is no significant difference between the solutions of the system if the spores come from the brood or from the cell comb. The fate of the colony will be the same. On the other hand, if the initial condition of the system is (0,0,0,0,10000,1) (see Fig 6), which means that there is a spore-carrier adult bee carrying sufficient number of spores that can start brood infection, the colony will also die out. However, the colony takes longer time to die out compared with the result where the initial condition of the system is either (0,0,1,0,10000,0) or (0,0,0,1,10000,0) (see Figs 4 or 5).

### The case when there is a constant rate of contamination from the foraging area (*α*_4_ ≠ 0)

**Fig 7:**
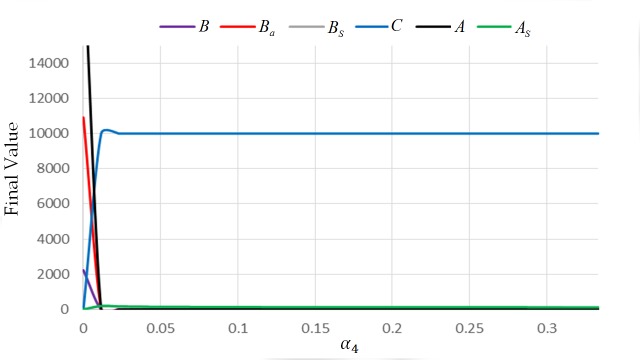
Final values of the state variable with different values of *α*_4_: initial condition (0,0,0,0,10000,0) and parameters *L* = 1500, *w* = 21000, *v* = 10000, *α*_3_ = 0.0005, *α*_2_ = 1, *α*_1_ = 0.001, *φ*_1_ = 1/3, *φ*_2_ = 1/3 *β*_1_ = 1/3, *β*_2_ = 1/15, and *μ* = 1/30, End-Time = 200.

**Fig 8:**
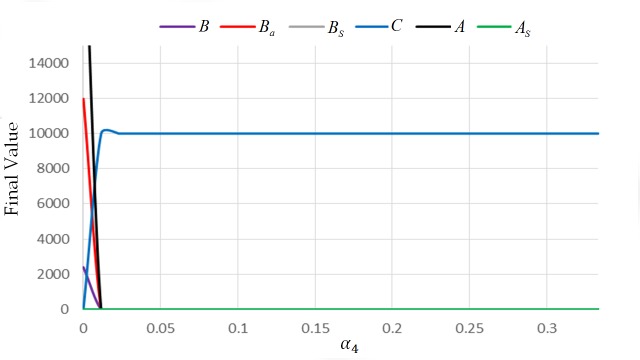
Final values of the state variable with different values of *α*_4_: initial condition (0,0,0,0,10000,0) and parameters *L* = 1500, *w* = 21000, *v* = 10000, *α*_3_ = 0.0005, *α*_2_ = 1, *α*_1_ = 0.001, *φ*_1_ = 1/3, *φ*_2_ = 1/3 *β*_1_ = 1/3, *β*_2_ = 1/15, and *μ* = 1/30, End-Time = 500.

**Fig 9:**
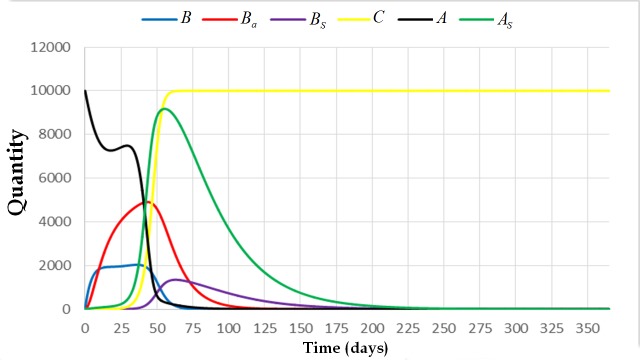
The trajectory of the system Eq (7) with initial condition (0,0,0,0,10000,0) and parameters *L* = 1500, *w* = 21000, *v* = 10000, *α*_4_ = 0.001, *α*_3_ = 0.0005, *α*_2_ = 1, *α*_1_ = 0.001, *φ*_1_ = 1/3, *φ*_2_ = 1, *σ* = 0.001, *β*_1_ = 1/5, *β*_2_ = 1/13, and *μ* = 1/30. This figure illustrates what will happen to the colony if the value of *α*_4_ (the rate at which adult bees become infected upon contact with the foraging area) is non-zero.

For this case, the extinction equilibrium state is non-hyperbolic since one column of the Jacobian matrix has zero column (see S1 File). To provide examples, we employed numerical results and observed the behavior of the system near the extinction equilibrium state (see Figs 7 and 8). From the simulations, all the solutions of the system tend to approach (0,0,0, C*,0,0). Specifically, all the solutions approach (0,0,0, *v*,0,0), where *v* is the maximum number of cell combs in the colony. This means that the colony will eventually die when it is exposed to AFB. Fig 9 illustrates what will happen to a colony if the value of *α*_4_ ≠ 0.

Clinical symptoms can be observed if the value of *C* is already greater than one. In the presence of clinical symptoms, the population of bees in the colony still escalates up to a certain time period. Then the population gradually declines (see Fig 9). For this case, the number of adult bees started to decline after 75 days. There is also an abrupt decline on the number of spore-free adult bees from the 50th day up to the 60th day of exposure. The number of broods also started to decline after 60 days. The colony collapses after 200 days. Moreover, the number of spore-free adult bees is decreasing and approaching to zero rapidly. At 75th day, this number already reached zero which means that all of the adult bees are already spore-carriers. At this moment, the colony is already collapsing since almost all of the cell combs are already contaminated with AFB. This can be observed by observing the trajectory of state variable *C*.

### Perturbation of Parameters

We varied the values of some of the parameters to determine their effects on the system. Simulations show that the parameter *σ* (the percentage of clean cell combs that will be contaminated with AFB spores) gives the big effect on shortening or lengthening the life of the colony compared to the other parameters. On the other hand, the parameter *α*_3_ (the rate at which adult bees become infected upon contact with spore-carrier adult bees) has minimal effect on the system. The parameters *α*_1_ (the rate at which broods are infected by spore-carrier adult bees), *α*_4_ (the rate at which adult bees become infected upon contact with the foraging area) and, *φ*_2_ (the rate at which a diseased dead brood will be cleaned by adult bees) have the same effect on shortening and lengthening the life of the colony. Hence, to control the spread of AFB, the parameters *σ*, *α*_1_, *α*_4_, and *φ*_2_ should be taken into consideration. However, based on the innate behavior of honeybees, the parameters *φ*_2_ and *σ* are difficult to control because honeybees clean their cell combs constantly. The only feasible parameter that can be managed is the parameter *α*_1_ which can be done by strengthening the immunity of larvae to spores.

## Discussion

Our model illustrates how AFB spores spread throughout a honeybee colony. The model aims to capture the long term fate of the colony once exposed to AFB spores. Without effective and efficient treatment, AFB infection will lead to colony collapse. The only way that a colony, in a natural condition, to be spore-free is by preventing AFB to enter the hive.

**Fig 10:**
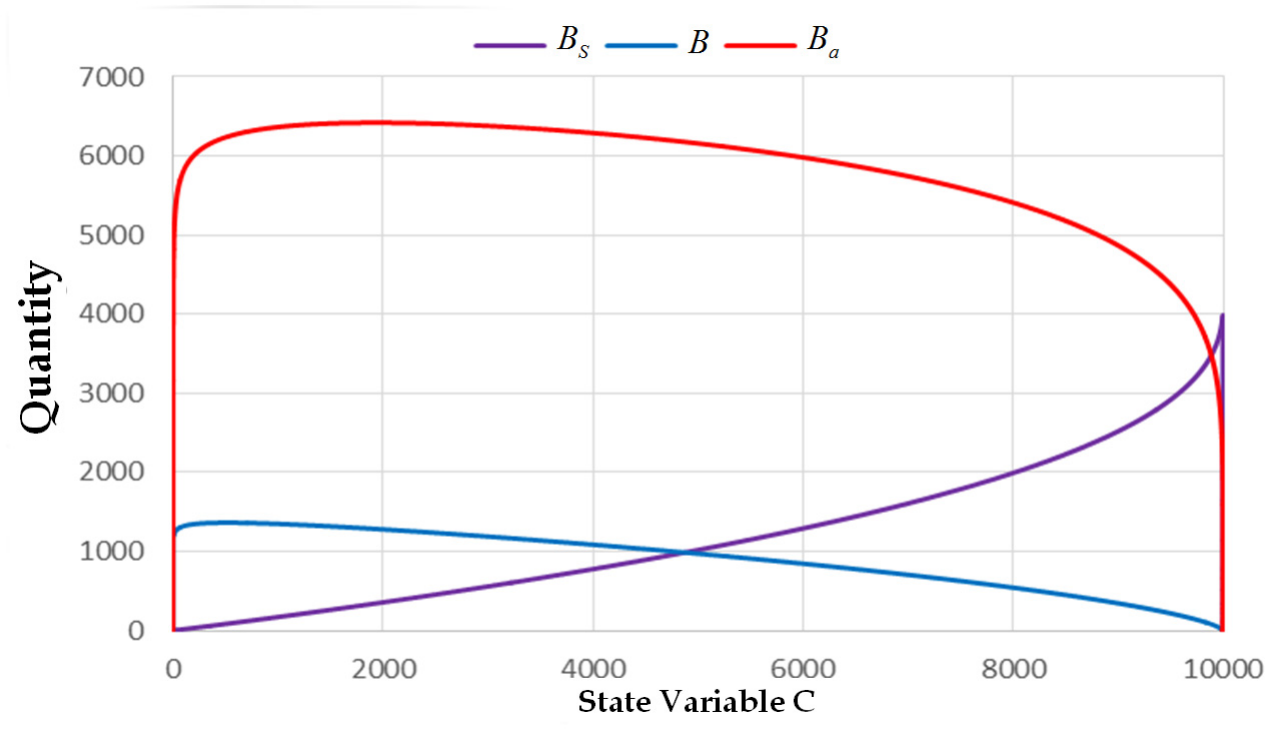
The trajectory of the state variable *B*, *B*_*a*_ and *B*_*s*_ versus the state variable *C* with initial condition (0,0,0,0,0,10000,0) and parameters. *L* = 1500, *w* = 21000, *v* = 10000, *α*_4_ = 1/3, *α*_3_ = 0.001, *α*_2_ = 1, *α*_1_ = 0.001, *φ*_1_ = 1/3, *φ*_2_ = 1/3, *σ* = 0.001, *β*_1_ = 1/3, *β*_2_ = 1/15, and *μ* = 1/30.

**Fig 11:**
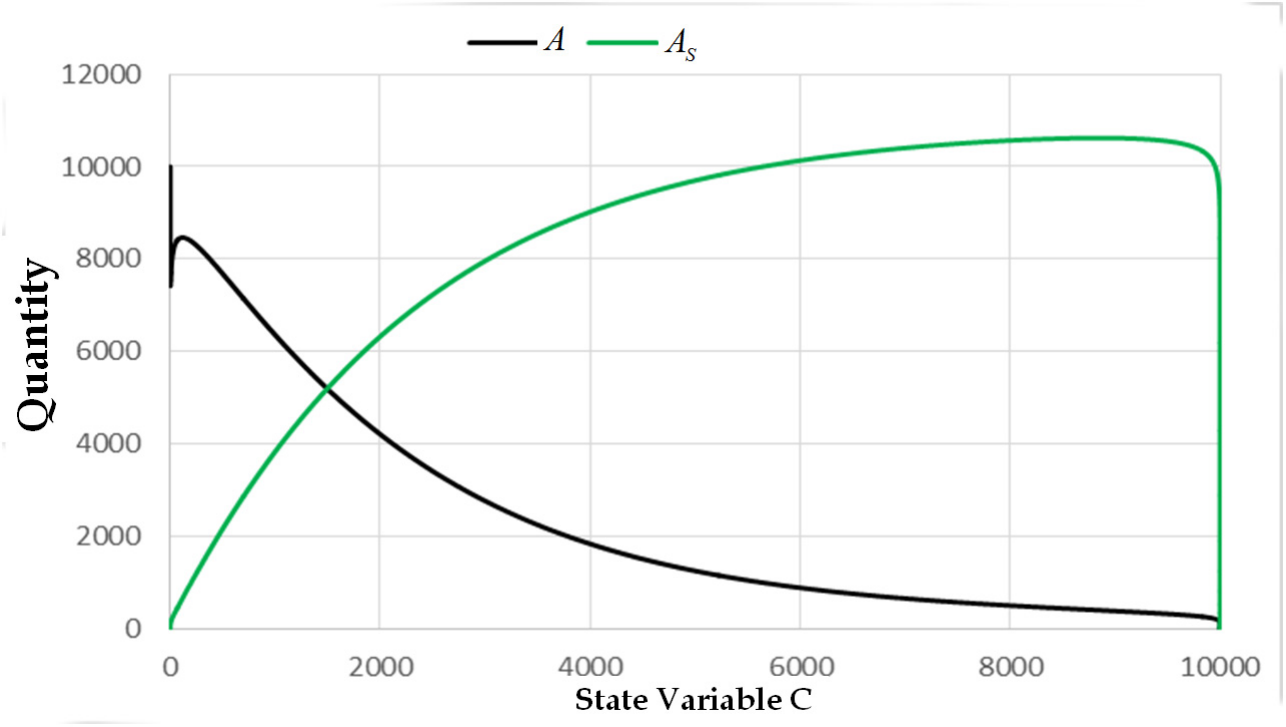
The trajectory of the state variable *A* and *A*_*s*_ versus the state variable *C* with initial condition (0,0,0,0,0,10000,0) and parameters. *L* = 1500, *w* = 21000, *v* = 10000, *α*_4_ = 1/3, *α*_3_ = 0.001, *α*_2_ = 1, *α*_1_ = 0.001, *φ*_1_ = 1/3, *φ*_2_ = 1/3, *σ* = 0.001, *β*_1_ = 1/3, *β*_2_ = 1/15, and *μ* = 1/30.

The next interesting result that needs emphasis in our model is how the number of infected cell combs, *C*, becomes one of the catalysts of the spread of AFB within the colony. We showed how the value of the state variable *C* affects the other state variables. We can observe (see Figs 10 and 11) that the values of *B*, *Ba*, *Bs*, *A*, and *As* approach zero as the value of *C* approaches 10000. Based on this observation, we can say that even with the clinical symptoms, the colony may be mistakenly seen to be a strong colony. This claim can also be seen in the previous figures, wherein the quantity of the adult bees grows up even with clinical symptoms until it reaches the time that it will decline rapidly.

The aforementioned observations can be used to further elucidate when will a given colony be eradicated once infected by AFB. The total loss of the adult bees happens if almost all of the cell combs are already infected by spores. Moreover, with only less than 500 infected cell combs in the colony, almost all of the adult bees are already spore-carriers. In addition to that, the number of adult bees started to decline if almost all of the cell combs are already contaminated. In fact, we can see in Fig 9 that the state variable *C* reaches its maximum value at the 55^th^ day of the exposure of the colony. We can also observe in the previous simulations that the state variable *C* approaches its maximum value very fast once its value is already equal to one. Once there is already a clinical symptom, the spores will spread throughout the cell comb in a very short span of time. The time of the spread can be influenced by the parameter *σ*.

From the results that we have generated, we come up with the following recommendations. The beekeepers should avoid keeping honeybees in places with known AFB outbreak. If unavoidable, it is better to focus on preventing spores from infecting the larvae of the colonies. This can be done by strengthening the immunity of the larvae from the possible infection of spores, such as by introducing probiotics and pollen feeding. For future study, we can consider adding or incorporating factors that strengthen the resistance of the larvae from the spores. This can help us evaluate the impact of the resistance in inhibiting the possible infection of the larvae.

## Supporting Information

S1 File Appendix: Derivation of the equilibrium states and their stability analysis. (PDF)
