# Supplementary material for: A Mathematical Model of Intra-Colony Spread of American Foulbrood in European Honeybees (Apis mellifera L.)
Source: PLoS One. 2015 Dec 16;10(12):e0143805. doi: 10.1371/journal.pone.0143805 (PMC4682658; doi:10.1371/journal.pone.0143805)
Supplement: S1 File — (PDF) [file pone.0143805.s001.pdf]

Appendix

The mathematical model of the spread of AFB in a controlled environment is given by the following system of ordinary differential equations:

$$\frac{dB}{dt} = M - \alpha_1 PB - \frac{C}{v}M - \beta_1 B \tag{1}$$
$$\frac{dB_a}{dt} = \beta_1 B - \beta_2 B_a \tag{2}$$
$$\frac{dB_s}{dt} = \alpha_1 PB + \frac{C}{v}M - \varphi_1\varphi_2 B_s \tag{3}$$
$$\frac{dC}{dt} = \varphi_1\varphi_2 B_s\left[\sigma\left(v - C\right)\right] \tag{4}$$
$$\frac{dA}{dt} = \beta_2\left(1 - \alpha_1 P\right)B_a - \alpha_2\frac{C}{v}A - \alpha_3 PA - \alpha_4 A - \mu A \tag{5}$$
$$\frac{dA_s}{dt} = \beta_2\alpha_1 PB_a + \alpha_2\frac{C}{v}A + \alpha_3 PA + \alpha_4 A - \mu A_s \tag{6}$$

where $P = \dfrac{A_s}{1 + A + A_s}$ and $M = L\dfrac{A + A_s}{w + A + A_s}$.

For simplicity, we write the system as

$$\frac{dX_i}{dt} = F_i(X), i = 1, 2, \cdots, 6 \tag{7}$$

where $X = (X_1, X_2, X_3, X_4, X_5, X_6) = (B, B_a, B_s, C, A, A_s)$.

Finding the Equilibrium Points

To find the equilibrium states of our system (7), we need to equate all $F_i(X)$ to zero for all $i = 1, 2, 3, 4, 5, 6$. Hence, we have the following:

$$L\frac{A + A_s}{w + A + A_s} - \alpha_1\frac{A_s}{1 + A + A_s}B - L\frac{C}{v}\frac{A + A_s}{w + A + A_s} - \beta_1 B = 0 \tag{8}$$
$$\beta_1 B - \beta_2 B_a = 0 \tag{9}$$
$$\alpha_1\frac{A_s}{1 + A + A_s}B + L\frac{C}{v}\frac{A + A_s}{w + A + A_s} - \varphi_1\varphi_2 B_s = 0 \tag{10}$$
$$\varphi_1\varphi_2 B_s\left[\sigma\left(v - C\right)\right] = 0 \tag{11}$$
$$\beta_2\left(1 - \alpha_1\frac{A_s}{1 + A + A_s}\right)B_a - \alpha_2\frac{C}{v}A - \alpha_3\frac{A_s}{1 + A + A_s}A - \alpha_4 A - \mu A = 0 \tag{12}$$
$$\beta_2\alpha_1\frac{A_s}{1 + A + A_s}B_a + \alpha_2\frac{C}{v}A + \alpha_3\frac{A_s}{1 + A + A_s}A + \alpha_4 A - \mu A_s = 0. \tag{13}$$

For the disease free equilibrium point, we set $B_s = C = A_s = 0$, so that the above equations become:

$$L\frac{A}{w + A} - \beta_1 B = 0 \tag{14}$$
$$\beta_1 B - \beta_2 B_a = 0 \tag{15}$$
$$\beta_2 B_a - \alpha_4 A - \mu A = 0 \tag{16}$$
$$\alpha_4 A = 0 \tag{17}$$

From (17), it will follow that $\alpha_4 = 0$ since A can not be equal to zero. This means that the disease-free equilibrium point will exist only if the rate at which adult bees will be infected upon contact to the infected food outside the hive is equal to zero.

From equations (14)−(16), we have,

$$\begin{aligned} &L\frac{A}{w+A} - \mu A = 0 \\ \Rightarrow &A\left(\frac{L}{w+A} - \mu\right) = 0 \\ \Rightarrow &\frac{L}{w+A} - \mu = 0, \text{ since } A \neq 0 \\ \Rightarrow &\frac{L}{w+A} = \mu \\ \Rightarrow &w + A = \frac{L}{\mu} \end{aligned}$$

$$\Rightarrow A = \frac{L}{\mu} - w \tag{18}$$

$$\Rightarrow A = \frac{L - w(\mu)}{\mu} \tag{19}$$

From equations (15) and (16) we have,

$$\begin{aligned} \mu A &= \beta_1 B \\ \Rightarrow B &= \frac{\mu A}{\beta_1} \end{aligned}$$

$$\Rightarrow B = \frac{L - w(\mu)}{\beta_1} \tag{20}$$

From equation (15) we have,

$$B_a = \frac{\beta_1 B}{\beta_2}$$

$$\Rightarrow B_a = \frac{L - w(\mu)}{\beta_2} \tag{21}$$

Thus, the disease free equilibrium point of the system (7) is given by $X^* = \left(\frac{L - w\mu}{\beta_1}, \frac{L - w\mu}{\beta_2}, 0, 0, \frac{L - w\mu}{\mu}, 0\right)$. Aside from the condition that $\alpha_4 = 0$, one thing that we need to consider so that X^* exists is that $\frac{L}{w\mu} > 1$ since $L - w\mu$ must be greater than zero.

From the given assumptions, the colony is asssumed to be healthy from the start. Thus, if $\alpha_4 = 0$ with initial condition $(B, B_a, 0, 0, A, 0)$, the system (7) can be reduced into system of ordinary differential equations of three state variables: A, B, B_a. The reduced system (22) is given by

$$\begin{aligned} \frac{dB}{dt} &= L\frac{A}{w+A} - \beta_1 B \\ \frac{dB_a}{dt} &= \beta_1 B - \beta_2 B_a \\ \frac{dA}{dt} &= \beta_2 B_a - \mu A \end{aligned} \tag{22}$$

It is more interesting to find out what will happen to our system (7) if $\alpha_4 \neq 0$. In this case, it is more practical to study the behavior of the solution of the system (7) if $\frac{L}{w\mu} > 1$ since otherwise, the colony as we will see in Corollary (2) will die.

The next theorem will tell us that if $\alpha_4 \neq 0$ then the trivial equilibrium state and the spore-free equilibrium state X^* do not exist.

Theorem 1. *In system (7), if $\alpha_4 \neq 0$ then C assumes a positive value.*

Proof. From equation (4), we have

$$\frac{dC}{dt} = \varphi_1\varphi_2 B_s \left[\sigma\left(v - C\right)\right].$$

It is clear that the value of $\frac{dC}{dt}$ is always greater than or equal to zero depending on the the value of B_s. If $\alpha_4 \neq 0$, there is a moment that A_s is non-zero which means that B_s has also a moment that it is non-zero. Hence, C will be increasing and converging to a non-zero value. □

Theorem 2. *If the point $(0, 0, B_s, C, 0, A_s)$ is an equilibrium state of the system (7), then $A_s = B_s = 0$.*

Proof. If $B = 0, B_a = 0$ and $A = 0$ then

$$L\frac{A_s}{w + A_s} - \left(\frac{C}{v}\right)\left(\frac{A_s}{w + A_s}\right) = 0 \tag{23}$$

$$\left(\frac{C}{v}\right)\left(\frac{A_s}{w + A_s}\right) - \varphi_1\varphi_2 B_s = 0 \tag{24}$$

$$\varphi_1\varphi_2 B_s \left[\sigma\left(v - C\right)\right] = 0 \tag{25}$$

$$\mu A_s = 0 \tag{26}$$

In (26), $\mu = 0$ or $A_s = 0$. However, μ cannot be equal to zero since it is the death rate of adult bees. Hence, A_s will be forced to be equal to 0. If $A_s = 0$ then from (24) $\varphi_1\varphi_2 B_s = 0$. Since φ_1 and φ_2 cannot be equal to 0, then $B_s = 0$.

Therefore, the theorem holds. □

Note that in the proof of the previous theorem, C can be any value. This means that the point $(0, 0, 0, C, 0, 0)$, where $C \in \mathbb{R}^+$, is an equilibrium point of system (7). This equilibrium state occurs only when the value of the parameter $\alpha_4 \neq 0$.

In the next theorem, we will show that $(0, 0, 0, 0, 0, 0)$, X^* and $(0, 0, 0, C, 0, 0)$ are the only equilibrium states of the system (7).

Theorem 3. *The system (7) has only three equilibrium states: $(0, 0, 0, 0, 0, 0)$, X^* and $(0, 0, 0, C, 0, 0)$, where $C \in \mathbb{R}^+$.*

Proof. From equation (11), we have

$$\varphi_1\varphi_2 B_s \left[\sigma\left(v - C\right)\right] = 0.$$
$$\Rightarrow \varphi_1\varphi_2 B_s = 0 \text{ or } \sigma\left(v - C\right) = 0$$

Case 1: If $\varphi_1\varphi_2 B_s = 0$ and $C = 0$ then (10) will be $\alpha_1 \frac{A_s}{1 + A + A_s} B = 0$. There are two cases that can be considered in this equation: $B = 0$ or $A_s = 0$. If $B = 0$ then using Equation 9, $B_a = 0$. Substituting the values of $B_a = 0$ and $C = 0$, Equation 12 will be

$-\alpha_3 \dfrac{A_s}{1+A+A_s} A - \alpha_4 A - \mu A = 0$. This equation will give the value of $A = 0$. Hence, the equilibrium state that can be derived from this case will be the trivial equilibrium state. Now, for the second case, we will take the value of $A_s = 0$. If we substitute the values $C = 0$ and $A_s = 0$ to Equations 8−13 then we will have the following equations: $\beta_1 B - \beta_2 B_a = 0$, $\alpha_4 A = 0$, and $\beta_2 B_a - \alpha_4 - \mu A = 0$. From these equations, the disease free equilibrium state will be derived.

Case 2: If $\varphi_1\varphi_2 B_s = 0$ and $C \neq 0$ then $B_s = 0$ since $\varphi_1\varphi_2$ cannot take a zero value. Equation (10) will be $\alpha_1 \dfrac{A_s}{1+A+A_s} B + \dfrac{C}{v} M = 0$. Since the two terms are greater than or equal to zero then $\alpha_1 \dfrac{A_s}{1+A+A_s} B = 0$ and $L \dfrac{C}{v} \dfrac{A+A_s}{w+A+A_s} = 0$. Since $C \neq 0$ then A_s will be zero. Equation 13 will be $\alpha_2 \dfrac{C}{v} A + \alpha_4 A = 0$. This implies that $A = 0$, since $\alpha_4 \neq 0$ if $C \neq 0$. If $A = 0$ then in Equation 12, $B_a = 0$. This will make $B = 0$ in Equation 9. Hence, for this case the point $(0, 0, 0, C, 0, 0)$ is found to be one of the equilibrium points.

Case 3: If $\varphi_1\varphi_2 B_s \neq 0$ and $C \neq 0$ then $\sigma(v - C) = 0$.

$\Rightarrow v - C = 0$, since σ cannot be equal to zero.

$\Rightarrow C = v$.

Equation (8) will become $\alpha_1 \dfrac{A_s}{1+A+A_s} B + \beta_1 B = 0$

$\Rightarrow B = 0$ since the two terms on the left side of the above equation cannot be negative.

$\Rightarrow B_a = 0$, from Equation (9).

From Equations (12) and (13), $A = 0$ and $A_s = 0$

$\Rightarrow B_s = 0$, Using Equation (10)

$\Rightarrow C = v$.

Thus, for this case the point $(0, 0, 0, v, 0, 0)$ will be the equilibrium state.

Therefore, the theorem holds.

□

As a biological remark:

If the value of $C = v$ then all of the eggs laid by the queen will be surely infected. If this is the case, there will be no more eggs that will eclose to adult bees so that A_s and A will converge to zero.

From Theorems (1) and (3) we have the following conclusion.

Theorem 4. *In system (7), if* $\alpha_4 \neq 0$ *then the point* $(0, 0, 0, C, 0, 0)$, $C \in \mathbb{R}^+$ *is the only equilibrium state.*

Stability Analysis

To study the stability behavior of the equilibrium states, $(0, 0, 0, 0, 0, 0)$ and $X^* = \left(\dfrac{L-w\mu}{\beta_1}, \dfrac{L-w\mu}{\beta_2}, 0, 0, \dfrac{L-w\mu}{\mu}, 0\right)$, of system (7) we can just simply consider system (22). The equilibrium states $(0, 0, 0, 0, 0, 0)$ and X^* are equivalent to $(0, 0, 0)$ and $\left(\dfrac{L-w\mu}{\beta_1}, \dfrac{L-w\mu}{\beta_2}, \dfrac{L-w\mu}{\mu}\right)$ in system (22) respectively.

Theorem 5. *In system (22), if* $\dfrac{L}{w\mu} > 1$ *then the equilibrium state* $X^{**} = \left(\dfrac{L-w\mu}{\beta_1}, \dfrac{L-w\mu}{\beta_2}, \dfrac{L-w\mu}{\mu}\right)$ *is asymptotically stable.*

Proof. We will use the linearization method to show that the equilibrium state X^{**} is locally asymptotically stable when $\dfrac{L}{w\mu} > 1$.

The Jacobian matrix of system (22) at X^{**} is given by

$$JF\left(X^{**}\right) = \begin{pmatrix} -\beta_1 & 0 & \dfrac{Lw}{\left(w + \dfrac{L - w\mu}{\mu}\right)^2} \\ \beta_1 & -\beta_2 & 0 \\ 0 & \beta_2 & -\mu \end{pmatrix}$$

To show that the given equilibrium state is stable, we need to show that the real part of the eigenvalues of $JF\left(X^{**}\right)$ are all negative. Here, we will use the Routh-Hurwitz Criterion.

The characteristic polynomial of $JF\left(X^{**}\right)$ is $\lambda^3 + (\mu + \beta_1 + \beta_2)\lambda^2 + (\mu(\beta_1 + \beta_2) + \beta_1\beta_2)\lambda + \dfrac{1}{L}\mu\beta_1\beta_2(L - w\mu)$. Let $a_1 = \mu + \beta_1 + \beta_2$, $a_2 = \mu(\beta_2 + \beta_2) + \beta_1\beta_2$ and $a_3 = \dfrac{1}{L}\mu\beta_1\beta_2(L - w\mu)$. The real part of the eigenvalues are all negative if the following conditions are satisfied:

i.) $a_1 > 0$ and $a_3 > 0$

ii.) $a_1 a_2 > a_3$

It is clear that a_1 is always positive while a_3 is positive only if $\dfrac{L}{w\mu} > 1$. The last step that we need to show is that $a_1 a_2 > a_3$. Now,

$a_1 a_2 = \mu^2\beta_1 + \mu^2\beta_2 + \mu\beta_1^2 + 3\mu\beta_1\beta_2 + \mu\beta_2^2 + \beta_1^2\beta_2 + \beta_1\beta_2^2$ and it is clear that $a_1 a_2 > a_3$ since $\mu\beta_1\beta_2 > \frac{w\mu}{L}$ and $a_1 a_2 > 3\mu\beta_1\beta_2$. Thus, all the real parts of the eigenvalues of $JF\left(X^{**}\right)$ are negative. Therefore, the theorem holds. □

Theorem 6. *In system (22), if* $\dfrac{L}{w\mu} < 1$ *then the equilibrium state* $(0,0,0)$ *is asymptotically stable.*

Proof. The Jacobian matrix of system (22) at $(0,0,0)$ is given by

$$JF(0,0,0) = \begin{pmatrix} -\beta_1 & 0 & \dfrac{L}{w} \\ \beta_1 & -\beta_2 & 0 \\ 0 & \beta_2 & -\mu \end{pmatrix}.$$

Its characteristic polynomial is $\lambda^3 + (\mu + \beta_1 + \beta_2)\lambda^2 + (\mu(\beta_1 + \beta_2) + \beta_1\beta_2)\lambda + \left(\mu\beta_1\beta_2 - \dfrac{L}{\mu w}\beta_1\beta_2\right)$. Let $a_1 = \mu + \beta_1 + \beta_2$, $a_2 = \mu(\beta_2 + \beta_2) + \beta_1\beta_2$ and $a_3 = \left(\mu\beta_1\beta_2 - \dfrac{L}{\mu w}\beta_1\beta_2\right)$. a_1 is always positive while a_3 is positive only if $\dfrac{L}{w\mu} < 1$.

Observe that $a_1 a_2 = \mu^2\beta_1 + \mu^2\beta_2 + \mu\beta_1^2 + 3\mu\beta_1\beta_2 + \mu\beta_2^2 + \beta_1^2\beta_2 + \beta_1\beta_2^2$ and it is clear that $a_1 a_2 > a_3$ since $\mu\beta_1\beta_2 > \dfrac{L}{\mu w}\beta_1\beta_2$ and $a_1 a_2 > 3\mu\beta_1\beta_2$. Thus, all the real part of the eigenvalues of $Jf(0,0,0)$ are negative. Therefore, the theorem holds. □

Corollary 1. *In system (7) with initial condition* $(B, B_a, 0, 0, A, 0)$*, if* $\alpha_4 = 0$ *and* $\dfrac{L}{w\mu} > 1$ *then* X^* *is asymptotically stable.*

Corollary 2. *In system (7) with initial condition* $(B, B_a, 0, 0, A, 0)$*, if* $\alpha_4 = 0$ *and* $\dfrac{L}{w\mu} < 1$ *then* $(0,0,0,0,0,0)$ *is asymptotically stable.*

The two corollaries above immediately follow from Theorems 5 and 6. From Corollary 1, we can conclude that the colony will be free of AFB spores if $\alpha_4 = 0$. Moreover, the colony will survive if the value of $\dfrac{L}{w\mu} > 1$. Here, $\dfrac{L}{w\mu}$ serves as the threshold of the system (7) if $\alpha_4 = 0$.

If $\dfrac{L}{w\mu} = 1$ then X^* and $(0,0,0,0,0,0)$ are non-hyperbolic equilibrium states. Hence, linearization fails to show that they are asymptotically stable.

Remark 1. *In the initial condition of system (7), if the values of either* B_s, C_w, C*, and* A_s *have non-zero value then the equilibrium states* $(0,0,0,0,0,0)$ *and* X^* *are unstable.*

Theorem 7. *The equilibrium state* $(0,0,0,C,0,0)$*, where* C *is non-zero, is non-hyperbolic.*

Proof. The Jacobian matrix of the system (7) at $(0,0,0,C,0,0)$ is given by
$JF(0,0,0,C,0,0) =$

$$\begin{pmatrix} -\beta_1 & 0 & 0 & 0 & \dfrac{L}{w} - \dfrac{LC}{vw} & \dfrac{L}{w} - \dfrac{LC}{vw} \\ \beta_1 & -\beta_2 & 0 & 0 & 0 & 0 \\ 0 & 0 & -\varphi_1\varphi_2 & 0 & \dfrac{LC}{vw} & \dfrac{LC}{vw} \\ 0 & 0 & \varphi_1\varphi_2\sigma v - \varphi_1\varphi_2\sigma C & 0 & 0 & 0 \\ 0 & \beta_2 & 0 & 0 & -\alpha_2\dfrac{C}{v} - \alpha_4 - \mu & 0 \\ 0 & 0 & 0 & 0 & \alpha_2\dfrac{C}{v} + \alpha_4 & -\mu \end{pmatrix}.$$

We can observe that one of the columns of $JF(0,0,0,C,0,0)$ is a zero vector. From this, we can say that one of its eigenvalues will be equal to zero. This makes the equilibrium state $(0,0,0,C,0,0)$ non-hyperbolic. □
